# Women’s perspectives of molecular breast imaging: a qualitative study

**DOI:** 10.1038/s41416-024-02930-1

**Published:** 2024-12-18

**Authors:** Helen Elliott, A. Joy Allen, Nerys D. Forester, Sara Graziadio, W. S. Jones, Beverley Clare Lendrem, Mark S. Pearce, Timothy Powell, Jason Scott, Alison Bray

**Affiliations:** 1 Royal Victoria Infirmary, Newcastle upon Tyne Hospitals NHS Foundation Trust, Newcastle upon Tyne, UK; 2 Translational and Clinical Research Institute, Newcastle University, Newcastle upon Tyne, UK; 3 NIHR Newcastle In Vitro Diagnostics Co-operative, Newcastle upon Tyne, UK; 4 Population Health Sciences Institute, Newcastle University, Newcastle upon Tyne, UK; 5 Department of Social Work, Education and Community Wellbeing, Northumbria University, Newcastle upon Tyne, UK

**Keywords:** Breast cancer, Population screening, Preclinical research, Molecular imaging, Molecular medicine

## Abstract

**Background:**

Mammography has poor sensitivity in dense breast tissue. Retrospective studies suggest that Molecular Breast Imaging (MBI), has superior diagnostic accuracy to mammography in women with very dense breast tissue. Women’s perspectives of MBI are unknown, but are crucial to understanding the feasibility of, and routes to, adoption into practice.

**Method:**

Semi-structured interviews with screened and unscreened women explored acceptability of MBI. Data were analysed thematically.

**Results:**

Four themes were generated from nineteen interviews: (1) presumed negative aspects of MBI are acceptable (2) convenience of access, (3) comfort in familiarity and (4) need for shared decisions relating to risk. Presumed negative aspects of MBI, such as radiation dose and forty-minute scan time, were acceptable provided there are benefits. Some participants were concerned about equitable access, such as parking. Participants expressed comfort in existing and familiar screening processes. Participants acknowledged that informing women of their breast density may result in increased anxiety, but it was still felt to be important to ensure women are fully informed of the risks and harms of screening.

**Conclusions:**

Women consider MBI to be an acceptable breast imaging modality. High-quality information enabling informed decision-making is essential.

## Background

Every year there are over 55,000 new incidences of breast cancer in the UK, and over 11,500 women die from breast cancer [1]. Cancer screening programmes aim to detect life-threatening tumours early, reduce future treatment costs, and ultimately reduce mortality. The National Health Service Breast Screening Programme (NHSBSP) comprises two population breast screening programmes: routine screening of women aged 50–70 every three years with mammography, and screening of women deemed at very high risk of breast cancer (with frequency and modality dependent on particular risk factors).

Breast cancer imaging is used in the UK in several interlinked pathways including the NHSBSP, such as symptomatic breast imaging, disease-response imaging and post-cancer surveillance imaging. Mammography is the primary imaging tool, with targeted ultrasound (USS) used to guide biopsy of suspicious lesions and Magnetic Resonance Imaging (MRI), the more sensitive test, limited to specific subsets of women, where it can be most effective, mainly due to capacity issues in the National Health Service (NHS).

Mammography relies on a difference between the density of a tumour and the surrounding normal breast tissue, causing tumours to be masked in women with dense breast tissue [2]. Recent evidence suggests that supplemental imaging should be offered to women with dense breast tissue due to the low sensitivity of mammography and independently increased risk of breast cancer in these women [3]. This is supported by a recent change in EU guidelines recommending that women be informed of their breast density to enable shared decisions about supplemental screening based on risk, and that those with very dense tissue should be offered additional breast MRI [4].

Molecular Breast Imaging (MBI) is a nuclear medicine breast imaging technique in use in the USA [5] in women with dense breast tissue. Retrospective studies have shown MBI to have superior diagnostic accuracy compared to mammography in this population [6, 7]. Whilst this provides compelling evidence for incorporating MBI into UK breast imaging pathway(s), no research has yet examined whether this would be acceptable to stakeholders, including patients, and what considerations would be required for its optimal deployment.

This study aimed to address this evidence gap by assessing the potential demand for MBI and perceived benefits if incorporated into the UK as a breast-imaging tool. The potential target population could include the current breast screening population, a subset thereof, for example, women identified as having large amounts of dense breast tissue on initial screening, and/or younger women at increased risk of breast cancer due to family history.

## Methods

### Participants, sampling, and recruitment

A combined purposive and snowball sampling strategy allowed selection of participants based on experience of breast cancer and/or screening. A pragmatic sample size of twenty participants was chosen after consultation with experienced qualitative researchers and considering the information power of the sample [8]. Participants were recruited by breast radiography staff while attending their routine mammogram and through social media adverts. Women over eighteen were invited. Initially only women with screening experience were invited, but after recommendation from patient representatives, this was expanded to include younger women who are more likely to be faced with a new, personalised breast screening programme.

### Data collection

Qualitative, semi-structured, exploratory, single-participant interviews were a mixture of face-to-face and online. Interviews, based on an iteratively designed topic guide, focused on individual patient stories, their journeys within breast screening, understanding of topics such as breast density, and opinions of MBI. Interview topic guides were developed in collaboration with patient representatives and Patient and Public Involvement panels, were tested in two pilot interviews and were updated after each interview as necessary to allow for relevant data collection. Background information about the NHSBSP was presented to help participants understand the potential setting of the new test and the background to the problem within breast screening that the new test is intended to address. Two animations, developed in collaboration with Kromek (Sedgefield, County Durham, UK), were included within the presentation. The first showed the mammogram patient journey (https://youtu.be/u1pQWwvACaI), and the second showed an equivalent proposed journey when attending for an MBI test (https://youtu.be/6nXEFkgEan0).

The lead researcher, HE, is a female below NHSBSP age with eight years of NHS work in patient-facing cancer-specific research. Other team members included AB, a clinical scientist, female, aged below NHSBSP age, with ten years’ experience working on implementation of novel devices into NHS pathways. Neither had lived experience of breast cancer or breast cancer screening. Supporting analysis was JS, a male, Associate Professor and Chartered Psychologist, with extensive expertise in health and social care systems research, including qualitative and mixed methods research.

### Data analysis

Data were audio-recorded and transcribed by a transcription company. Data were coded inductively within Nvivo 12 and analysed thematically using Braun and Clarke’s [9] six-stage process. HE maintained a reflexive diary throughout data collection with notes consulted during coding and incorporated into the analysis. During analysis, HE met regularly with both AB and JS to discuss themes and develop richer understandings of meaning.

## Results

Data collection was undertaken between January 2020 and July 2021. Nineteen participants were recruited and included in this analysis, of which 5 (26%) were face-to-face and 14 (74%) were held online. The shortest interview was 37 min, the longest was 1 h and 47 min, with a mean of 1 h and standard deviation of 16 min. Table 1 presents participant demographics, including age, location, and originating patient population.

**Table 1:** Participant demographics.

	Number of participants (percentage of sample)
Total	19 (100%)
Patient population	
Routine screening population stratified for dense breasts	8 (42%)
Higher risk women, either to replace MRI or in addition to MRI where used in dense breasts.	3 (16%)
Post cancer treatment surveillance stratified for dense breasts or under a certain age	2 (11%)
Younger than screening age	6 (32%)
Age range (mean = 49)	
18–39 (mean = 35)	5 (26%)
40–49 (mean = 45)	5 (26%)
50–69 (mean = 56)	7 (37%)
Over 70 (mean = 73)	2 (11%)
Urban or rural (self-declared)	
Urban	9 (47%)
Rural	4 (21%)
Not stated	6 (32%)
Participant has experienced breast screening?	
Yes	11 (58%)
No	8 (42%)

Four themes were constructed from the analysis:

1. Presumed negative aspects of MBI are acceptable.
2. Convenience of access.
3. Comfort in familiarity.
4. Need for shared decisions relating to risk.

Feedback was varied, but centred around radiation dose, imaging time and addition of a biopsy function. Participants also discussed mobile MBI scans, administration of the radioactive tracer using cannulation, and information requirements.

### Theme 1: Presumed negative aspects of MBI are acceptable

The whole-body effective dose of MBI is higher than that of mammography, with an associated increased risk of radiation-induced cancer. The current MBI scan time is 40 min, resulting in about an hour of clinic time, including cannulation and positioning. These aspects were explained to and discussed with all participants.

Participants were tolerant of both radiation dose, ‘I don’t think the extra risk would concern me from a radiation point of view’ (P006), and scan time, ‘I know there’s a bit longer wait, but, you know, neither here nor there [not important], really.’ (P001). Attitudes to radiation dose were related to feelings of trust in the NHS, ‘I would think that most people accept that the radiation dose that they were using was what was needed to do it in the best amount of time.’ (P013), and acceptance of increased risk given an increased benefit of an additional, more accurate test, ‘Your risk increases slightly [with MBI]. You are going to be there for a bit longer. But if it ends up being more successful in detecting that you either do or don’t have some breast cancer, then surely that’s okay. That would be fine with me.’ (P005).

Participants, when questioned about radiation levels, conveyed trust in the NHS system to decide appropriate levels of exposure, and expressed a need for information to better-understand risks and benefits. This idea is explored further in *Theme 4*.

Participants suggested that injections and cannulation are usually well-tolerated except by those with needle phobias. The additional burden of cannulation may be more acceptable if there was a clear benefit explained, again related to participants assessing risk and benefit (Theme 4*):* ‘I guess I’m at the stage where, maybe, this would be useful for me, because there would be a reason that I would need this, and maybe, a cannula wouldn’t be such a bad thing.’ (P012).

### Theme 2: Convenience of access

Of the eleven participants with experience of mammography screening, three attended mobile vans, seven attended a main hospital site, and one gave no information regarding this. The feeling among these participants was that although mobile screening offers quick, accessible appointments, they did not mind attending a hospital site because the perceived benefit of a screening scan outweighed this inconvenience. There is evidence in our data that women are keen to understand the risks and benefits of offered healthcare interventions and will tolerate inconvenience if there is a clear benefit (Theme 4*)*. One lady, who normally attends for mobile screening, does not mind her appointment being moved to a main hospital site, and said: ‘[sometimes] I’ve had to change my appointment, and I have had to come [to hospital]. They [asked] if I minded coming to the [hospital site], which I don’t.’, (P001). Another participant talked about the risk of increasing age and its impact on her acceptance of inconvenience, ‘We’ve all got [constraints on our time], but I just think with health issues…, and especially when you’re at a certain age,… you can’t mess around with your health, can you? So, whether you’ve got to sit there for six hours or two hours or an hour or five minutes, you’ve got to do it.’ (P002).

Some participants raised the issue of accessing main hospital sites and parking, which could reduce accessibility for some patients and may raise concerns about health inequalities, ‘having to travel somewhere like Newcastle and having to park and things [can be difficult].’ (P013).

### Theme 3: Comfort in familiarity

The data suggest that participants looked for, and were more comfortable with, aspects of healthcare with which they were familiar, ‘so [MBI] wouldn’t be any more dangerous or have any more radiation than if you had a broken arm and had to have that x-rayed or something like that?’ P005, and ‘if you compare [MBI] with other things that we’re encountering every day, it’s not really that onerous, is it?’ (P002).

Familiarity was associated with mammograms, ‘you have got this huge history with mammograms, so people know about mammograms.’ (P008), injections, ‘I have to have injections regularly anyway. They don’t concern me.’ (P006), breast units and staff ‘I felt safe coming to him [a particular consultant] every year to be examined’ (P010), procedures and protocols, ‘If that was what you started with, I think everybody would just accept it … just as people got used to what the mammography involved. It just becomes routine.’ (P013), and medicines ‘The radiation part itself, [I have] absolutely no issue with it, I think it’s a perfectly reasonable test to have done. Like you say, it’s used in lots of different areas, so I would have no issue with it.’ (P011).

Although participants seemed to favour familiar aspects, with one patient representative suggesting MBI be renamed ‘gammography’, they were keen to be involved in research and see changes and improvements to services, ‘I always think that it’s for the benefit not necessarily of just yourself, but for clinical studies. That’s how they find things out … a benefit for everybody.’ (P013).

### Theme 4: The need for shared decisions relating to risk

This theme is split into three subthemes. The first covers shared decision making, the second, information requirements, and the third, patients incorporating risk analyses in their decision-making.

#### Shared decision making

Many participants in this study had undergone screening themselves and so could be considered ‘pro-screening’, ‘I’m all for screening, I always take up any screening’ (P008), the feeling was one of trust in the system, ‘I have 100% trust in our medical people’ (P014). Participants felt that a test like MBI would not be offered by the NHS it if was not a ‘safe’ test, ‘I don’t believe anyone is going to inject something into me that they don’t believe is safe.’ (P009). They trusted clinicians and policymakers to fully investigate the harms and benefits and only offer the test if the latter outweighed the former, ‘They’re not going to just spend money on testing people willy-nilly [sic] for the sake of it.’ (P008). When harms associated with screening were discussed with participants, they were either unaware of or uninfluenced by them, ‘I personally don’t feel it [cancer screening] gets done frequently enough, but then I know you guys will be working on stats and that sort of thing. So, there are reasons that you do it [every three years], but from an anxiety type perspective I don’t feel like it gets done frequently enough.’ (P009) and remained in favour of screening for cancers.

There is strong evidence in the data that women want and need information to make their own decisions about risks and benefits, ‘Yes, and I think being informed with the risks of everything [is good], that’s an informed consent, isn’t it?’ (P003), and ‘I think if you’ve got all the information then you’ve got nothing to worry about.’ (P016).

#### Information requirements

All participants mentioned a desire for more information, despite making different decisions based on the content of the information. People described a need for information about breast density, ‘breast density, that’s not anywhere. You don’t read about it, you don’t hear about it.’ (P011), self-examination, detailed systematic information about potential tests, and the pathway itself, ‘I do like the steps, I like to know the steps and how we get there. And how long it takes at each of the stages, that was good to know … Is there anything afterwards I need to be aware of? But I think the video [study animations video(s)] generally was very clear at explaining everything that was going to happen.’ (P007). One participant highlighted the need for personalised information, especially when discussing risk, ‘I don’t think as an advertising … scheme just to put it [breast density information] out there, because I think it might scare people if they don’t know if they’ve got dense breast tissue or not. I think it’s probably dealt better with, on a one-to-one basis when they come in for their screening.’ (P014).

There was some suggestion in the data that informing women of their breast density may increase anxiety, ‘Probably a lot of people don’t know a lot about [breast density] … You don’t want to scare people about it, but I think everybody needs to be aware of it.’ (P006); despite this it was felt that this information was something women would like to be told, ‘I think so. I think it prepares people. Yes, I do. I think the more information… And they can digest it, look at it’ (P010). One participant suggested that if women are told they have high breast density, they might ‘self-examine’ more thoroughly and attend the GP more readily if they suspected changes in their breasts, ‘if more people knew that [about breast density], you know, they might be a little bit more pro-active with their own self-care opposed to being a little bit more lapsed with it or take a bit more of an interest in it … I don’t think people take enough control of it, but maybe if they were aware of the potential risks [of developing breast cancer] from that perspective they would.’ (P009). This raises questions around who could inform women about their breast density and whether the NHS has the resource to do this in busy screening clinics.

#### Patients undertaking risk benefit analyses in their decision making

Most women, when discussing current breast cancer screening as well as considering any new tests, talked in terms of the risks and benefits. There was evidence that participants were constantly weighing one against the other when deciding if this was something acceptable to them or not, ‘I guess that is what I was wondering, what’s the cost benefit [harm/benefit] of that [MBI]? Do you know if it’s more likely to find something and not to increase your … risk of harm massively in relation to what you would have as a routine thing anyway … then that sounds fair enough.’ (P005).

One participant suggested that MBI may be more suitable for patients with an increased risk of developing breast cancer, ‘Is there a thought that this type of test would be better for specific types of people? Would it be people … with denser breasts who maybe were higher risk, if they had a family history of it? Is that the thought that it would go down that route to begin with to see how well it identifies?’ (P005).

Participants were in favour of moving towards more personalised breast screening including individualised risk assessments, ‘I think it’s probably a smarter way of looking at screening. If you’re tailor-making screening to suit that individual person, it can only be of benefit to more individuals.’ (P007).

One participant felt that reducing screening for those at lower risk could be beneficial and might improve self-examination, ‘[re. reducing screening for those at ‘low risk’] I think you would still have your own concerns about your own breasts. Let’s say I was told, “You’ve got perfect breasts at the moment, we don’t need to see you for five years,” you’d still know to look out for dimples or swellings or hotspots or lumps or anything. You would still have those tools yourself. And if you had any concerns in that time period, you would raise them with your GP. I just think it is about that being proactive with your own body and doing your own self-assessment, as well as following any programmes that are available.’ (P007), and that it might be helpful to provide visual information for women to watch annually, ‘it could be that you go to that first screening and they say, “Right, we don’t need to see you for five years, but here is a little video,” and it could just be a simple link to a YouTube clip, that’s a bit cartoonified [sic], like that, but is actually very, very basic, showing, “This is what a hotspot looks like, this is what normal dimples look like, this where you should be checking.” So, they could be given a link, to say, “Watch this every year, [put this on your computer]… and make sure you’re doing this on a regular basis.” So, even if it’s just more information given at one point.’ (P007).

## Discussion

**Fig. 1:**
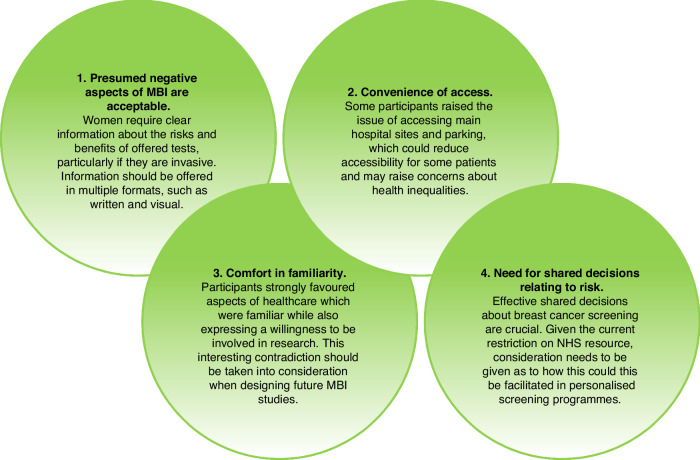
Overview of the main themes and resulting implications from this work. The four domains in the figure relate to the main themes and implications from this work and are based on the hypothetical incorporation of Molecular Breast Imaging into the breast cancer screening pathway as an adjunct to mammography in women with dense breast tissue.

This study is the first to examine women’s perspectives of incorporating MBI into the breast cancer screening pathway in the UK. There is work ongoing in the USA trialling lower dose and time imaging protocols to improve acceptability of MBI due to the perception that patients would find current dose and time protocols unacceptable [7, 10]. However, we found participants to be accepting of the presumed negative aspects of MBI, provided there was an associated benefit. An overview of the implications of this work is show in Fig. 1.

### Patient self-assessment of risk

Participants suggested they would be more accepting of invasive screening tests, such as MBI, if they perceive themselves to be at higher risk of developing breast cancer, due to age, for example. Harvie et al. [11] found similar patient self-assessment of risk in their study investigating retention in a weight loss programme aimed at reducing risk of breast cancer. Those participants who were told they were at an increased risk of developing breast cancer were significantly more likely to remain in the programme for more than twelve months. Similarly, Evans et al. [12] found, when informing patients of their individual risk of developing breast cancer as part of the PROCAS trial, that 99% of high-risk patients reattended for subsequent screening compared to 81% of low-risk patients. Therefore, we recommend that women invited for MBI be offered clear information explaining why a more invasive test is being offered to them and what benefits that test might offer.

### Patient Information and shared decisions

National cancer screening programmes require maximum uptake among eligible people to detect cancers, because large numbers of people need to be screened to detect relatively small numbers of cancers and thus save lives. For example, in the NHSBSP, 5.7 lives are saved per 1000 women screened [13]. National targets exist for all breast screening units in the UK, including an expected uptake among eligible people of at least 70% [14]. This means that patient information campaigns about screening have focused on the benefits of cancer screening to maximise uptake. There is a risk that this may be at the expense of effective shared decisions about the harms as well as the benefits of screening. Harms associated with cancer screening include radiation dose, potential overdiagnosis, and risk of false-positive results, associated extra workload such as biopsies, and patient anxiety [15].

Some participants conveyed a simple trust in the NHS to protect them from harmful amounts of radiation, relating to paternalistic medicine. Paternalism is seen as an old-fashioned term describing a patient-doctor construct, where the ‘principles of good medical care override individual treatment preferences’ [16] (p.41). Current medical healthcare promotes shared decision-making approaches to medical treatments, wherein patients are involved in decisions about their treatment with their doctor and medical team, based upon all parties being fully informed of associated benefits and harms [17]. There is evidence of paternalistic medicine in our data, which raises questions about whether shared decision-making is occurring effectively in a screening environment. This is increasingly important as we make a national move towards more personalised screening approaches, which could see low-risk women receiving less screening and higher-risk women offered more and different screening tests. Bartholomew et al. [18] advised that improvements are needed when facilitating decision-making for women offered breast cancer screening due to harms associated with overdiagnosis and false positives. They argued that offering incentives encouraging women to attend for screening is unethical and that instead women should be fully informed of all harms. This is also highlighted in the updated EUSOBI guideline [4], with emphasis placed on effective decision-making with women fully informed of the harms and risks associated with both breast screening and being diagnosed with dense breast tissue [4].

The literature sent to women who are eligible to be screened as part of the NHSBSP does include information on the harms of screening [19], but none of the women we spoke to discussed this. Perhaps the information is not clear enough in the written documentation, or perhaps women are not reading all of the documentation sent to them; either way this information is not being adequately communicated. In their US-based study, Austin et al. [20] found that informing women of their breast density did not increase awareness of the associated risk unless this was discussed directly with a caregiver. Raffle and Gray [21] emphasise that any screening programme is an ‘offer’ of a screening test. This implies that shared decision-making should be an important consideration and care should be taken to ensure that women who attend are fully aware of associated risks. Given that the success of a population screening programme includes maximising uptake, these two aspects are contradictory. It would be almost impossible to ensure that every woman invited for breast screening makes a fully informed, shared decision about her screening, given the current issue regarding resource of staff time in the NHS [22]. Some of our participants suggested that patient communications could be provided in formats other than written information, to aid ease of understanding. They commented that they would like to see information about screening in the same format as our study videos (link to the videos in the methods section), which outlined two patient journeys. One of the pillars of effective shared decision-making is high-quality patient information, and there is strong evidence within our data that women require comprehensible information in order to make decisions and feel as comfortable as possible about healthcare procedures. We therefore recommend that improved, regular, and up-to-date patient information should be available about breast density, self-examination, with detailed step-by-step information about potential tests and the screening pathway to allow women to make fully informed, shared decisions about their screening choices. Information should be available in different formats including videos as well as written documents.

Patients with extremely dense breast tissue (BI-RADS D) have a fourfold increased lifetime risk of developing breast cancer when compared to those with fatty breast tissue (BI-RADS A) [3]. Questions are being asked nationally and internationally through trials, such as the BRAID trial [23], about whether we should be offering enhanced risk-stratified screening programmes regardless of the reason for the risk, for example, increased breast density or family history. Patients with genetic abnormalities are offered extensive counselling along with supplemental screening, which is possible due to the low numbers of women falling into this category [14]. Shared decisions are made with these women to understand, firstly, if they want to know about their genetic status and secondly, if they want enhanced screening. If equivalent enhanced screening is offered to women with extremely dense breast tissue, do ethical obligations compel the NHS to offer equivalent counselling to these women too? If so, how can the NHS cope with the increased demand on resource, and who would undertake shared decisions with these women, regarding supplemental screening, given that they are currently allocated 6-min appointments for their screening? As discussed, participants expressed a desire for both more information and the ability to make considered shared decisions about their individual risk. There is evidence within our data that women would like to have as much information as possible; they acknowledge that breast density information may result in increased anxiety, but still wish to have this information. We recommend that consideration be given when planning personalised screening programmes, as to how best to deliver this information to women given current restrictions on resources, such as staff time. Further research could consider the impact on NHS resource of informing women of the associated increased risk of developing breast cancer and to consider ethically what level of risk counselling should be offered to women who have extremely dense breast tissue.

### Health inequalities

Between 2007/8 and 2012/13, Douglas et al. [24] found that there were socioeconomic differences in breast and cervical screening coverage between the extremes of income deprivation: in 2012/13 the most deprived quintile reached 69.8% screening coverage compared to 78.1% in the least deprived quintile. Over the five years of the study, inequalities between the two extreme groups remained, although the gap decreased, with coverage in the most deprived quintile rising from 66.3% to 69.8%. Interestingly, some of our participants raised concerns about the convenience of accessing supplemental imaging with MBI scans being at main hospital sites, which could have implications for the health inequality gap due to hidden costs associated with accessing healthcare [25]. Of course, this also applies to other hospital tests apart from mammography, most of which occur at main hospital sites. Certainly, the main competitors to MBI, such as MRI, are also likely to be based in the main sites rather than on mobile units. Changes to the screening programme should consider these potential health inequalities and ensure that those in the most deprived groups are not disadvantaged further and steps are taken to reduce this gap.

### Future study design

Although participants strongly favoured aspects of healthcare which were familiar, they also expressed a willingness to be involved in research and to see changes and improvements to services. This seems to be a conflict in the data that could be explained by a perceived benefit of access to new techniques. A qualitative study by Romo-Avilés et al. [26] involved discussions with patients about their reasons for taking part in a cardiovascular study. Hopes and expectations of participants made up one of four themes, with the other three relating to the specific medical condition. Several reasons for participants taking part in the study were grouped under the ‘hopes and expectations’ theme. Firstly, the invitation to take part coming from a trusted clinician, secondly, patient perception of receiving better care within a study, thirdly, a belief that participating in the study would give access to new interventions, and finally, altruism. This interesting contradiction between favouring familiarity and willingness to take part in clinical studies involving new interventions has implications when considering the recruitment strategy of a future MBI study and should be taken into consideration in the design phase of such a study.

### Strengths and limitations

Most interviews were conducted virtually due to the national decision to pause the breast screening programme due to the Covid-19 pandemic. This led to a delay in recruitment while the protocol was amended to allow recruitment from outside the screening programme to a virtual rather than face to face interview.

Although the overall effect of switching to virtual interviews increased flexibility for interviews and interviewees, the likelihood is that there is a cohort of non-computer literate participants who were inadvertently excluded, likely belonging to a lower socioeconomic demographic. The lead researcher (HE) reflexively felt it was also more difficult to develop rapport with participants online, which may have affected the richness of data collected in this way.

As mentioned in the methods section, one route recruiting women into the qualitative study was via a social media advert. One of the main findings of the study is that women seek as much information as possible, which could be due to the personality of this self-selecting group.

Also due in part to the recruitment strategy requiring participants to have access to online resources, participants possibly lacked socioeconomic diversity, as already mentioned. Aside from the lack of socioeconomic diversity, the sample also lacked ethnic and racial diversity, although the sample was diverse in relation to the age of participants and their experience of breast screening.

## Conclusion

Women consider MBI to be an acceptable breast imaging modality; they wish to be offered personalised, risk-based screening, with tests that offer favourable risk-benefit ratios. High-quality patient information enabling informed decision-making is essential. Further qualitative work is needed to understand how MBI will fit into existing screening pathways. There is also a need to understand the impact of informing women of their breast density and the associated increased risk of developing breast cancer.

## Supplementary information

COREQ Checklist

## Data Availability

The datasets generated and analysed during the current study are not publicly available due to legal frameworks protecting participant data, but anonymised quotes are available from the corresponding author on reasonable request.
