# Supplementary material for: Women’s perspectives of molecular breast imaging: a qualitative study
Source: Br J Cancer. 2024 Dec 18;132(3):276–82. doi: 10.1038/s41416-024-02930-1 (PMC11790831; doi:10.1038/s41416-024-02930-1)
Supplement: Supplementary file 1 — COREQ Checklist [file 41416_2024_2930_MOESM1_ESM.pdf]

1 Consolidated criteria for reporting qualitative studies (COREQ): 32-item checklist

1.1 Developed from:

Tong A, Sainsbury P, Craig J. Consolidated criteria for reporting qualitative research (COREQ): a 32-item checklist for interviews and focus groups. International Journal for Quality in Health Care. 2007;19(6):349-57.

No.	Item	Guide questions / description	Reported on page #
**Domain 1: Research team and reflexivity**			
**Personal Characteristics**			
1.	Interviewer / facilitator	Which author / s conducted the interview or focus group?	6
2.	Credentials	What were the researcher's credentials? *E.g. PhD, MD*	5-6
3.	Occupation	What was their occupation at the time of the study?	5-6
4.	Gender	Was the researcher male or female?	5-6
5.	Experience and training	What experience or training did the researcher have?	5-6
**Relationship with participants**			
6.	Relationship established	Was a relationship established prior to study commencement?	n/a
7.	Participant knowledge of the interviewer	What did the participants know about the researcher? *E.g. personal goals, reasons for doing the research*	5
8.	Interviewer characteristics	What characteristics were reported about the interviewer/facilitator? E.*g. Bias, assumptions, reasons and interests in the research topic*	5-6
**Domain 2: study design**			
**Theoretical framework**			
9.	Methodological orientation and Theory	What methodological orientation was stated to underpin the study? E.*g. grounded theory, discourse analysis, ethnography, phenomenology, content analysis*	6
**Participant selection**			
10.	Sampling	How were participants selected? E.*g. purposive, convenience, consecutive, snowball*	5
11.	Method of approach	How were participants approached? E.*g. face-to-face, telephone, mail, email*	5
12.	Sample size	How many participants were in the study?	6
13.	Non-participation	How many people refused to participate or dropped out?	none

		Reasons?	
**Setting**			
**14.**	Setting of data collection	Where was the data collected? *E.g. home, clinic, workplace*	5
**15.**	Presence of non-participants	Was anyone else present besides the participants and researchers?	5-6
**16.**	Description of sample	What are the important characteristics of the sample? *E.g. demographic data, date*	6, Table 1
**Data collection**			
**17.**	Interview guide	Were questions, prompts, guides provided by the authors? Was it pilot tested?	5
**18.**	Repeat interviews	Were repeat interviews carried out? If yes, how many?	n/a
**19.**	Audio / visual recording	Did the research use audio or visual recording to collect the data?	6
**20.**	Field notes	Were field notes made during and/or after the interview or focus group?	6
**21.**	Duration	What was the duration of the interviews or focus group?	6
**22.**	Data saturation	Was data saturation discussed?	5, information power was used.
**23.**	Transcripts returned	Were transcripts returned to participants for comment and/or correction?	n/a
**Domain 3: analysis and findings**			
**Data analysis**			
**24.**	Number of data coders	How many data coders coded the data?	6
**25.**	Description of the coding tree	Did authors provide a description of the coding tree?	No
**26.**	Derivation of themes	Were themes identified in advance or derived from the data?	6-7
**27.**	Software	What software, if applicable, was used to manage the data?	6
**28.**	Participant checking	Did participants provide feedback on the findings?	n/a
**Reporting**			
**29.**	Quotations presented	Were participant quotations presented to illustrate the themes / findings? Was each quotation identified? E.*g. participant number*	7-11
**30.**	Data and findings consistent	Was there consistency between the data presented and the findings?	7-15
**31.**	Clarity of major themes	Were major themes clearly presented in the findings?	7-15
**32.**	Clarity of minor themes	Is there a description of diverse cases or discussion of minor themes?	7-15
